# Potential for Genetic Improvement of Resistance to *Perkinsus olseni* in the Manila Clam, *Ruditapes philippinarum*, Using DNA Parentage Assignment and Mass Spawning

**DOI:** 10.3389/fvets.2020.579840

**Published:** 2020-10-22

**Authors:** Morgan Smits, Florian Enez, Serena Ferraresso, Giulia Dalla Rovere, Emilie Vetois, Jean-François Auvray, Lucie Genestout, Rachid Mahla, Giuseppe Arcangeli, Christine Paillard, Pierrick Haffray, Luca Bargelloni

**Affiliations:** ^1^Department of Comparative Biomedicine and Food Science (BCA), University of Padova, Legnaro, Italy; ^2^Syndicat des Sélectionneurs Avicoles et Aquacoles Français (SYSAAF), Laboratoire de Physiologie et Génomique des Poissons (LPGP), Campus de Beaulieu, Rennes, France; ^3^Société Atlantique de Mariculture (SATMAR), Gatteville-Phare, France; ^4^Labogena, Domaine de Vilvert, Jouy en Josas, France; ^5^National Reference Centre for Fish, Crustacean and Mollusc Pathology, Italian Health Authority and Research Organization for Animal Health and Food Safety (IZSVe), Legnaro, Italy; ^6^Laboratory of Marine Environmental Sciences (LEMAR), Institut Universitaire Européen de la Mer, Plouzané, France

**Keywords:** Manila clam (*Ruditapes philippinarum*), *Perkinsus olseni* infection, disease resistance, genetic selection, genetic parameter estimation

## Abstract

The Manila clam *Ruditapes philippinarum*, a major cultured shellfish species, is threatened by infection with the microparasite *Perkinsus olseni*, whose prevalence increases with high water temperatures. Under the current trend of climate change, the already severe effects of this parasitic infection might rapidly increase the frequency of mass mortality events. Treating infectious diseases in bivalves is notoriously problematic, therefore selective breeding for resistance represents a key strategy for mitigating the negative impact of pathogens. A crucial step in initiating selective breeding is the estimation of genetic parameters for traits of interest, which relies on the ability to record parentage and accurate phenotypes in a large number of individuals. Here, to estimate the heritability of resistance against *P. olseni*, a field experiment mirroring conditions in industrial clam production was set up, a genomic tool was developed for parentage assignment, and parasite load was determined through quantitative PCR. A mixed-family cohort of potentially 1,479 clam families was produced in a hatchery by mass spawning of 53 dams and 57 sires. The progenies were seeded in a commercial clam production area in the Venice lagoon, Italy, where high prevalence of *P. olseni* had previously been reported. Growth and parasite load were monitored every month and, after 1 year, more than 1,000 individuals were collected for DNA samples and phenotype recording. A pooled sequencing approach was carried out using DNA samples from the hatchery broodstock and from a Venice lagoon clam population, providing candidate markers used to develop a 245-SNP panel. Parentage assignment for 246 F1 individuals showed sire and dam representation were high (75 and 85%, respectively), indicating a very limited risk of inbreeding. Moderate heritability (0.23 ± 0.11–0.35 ± 0.13) was estimated for growth traits (shell length, shell weight, total weight), while parasite load showed high heritability, estimated at 0.51 ± 0.20. No significant genetic correlations were found between growth-associated traits and parasite load. Overall, the preliminary results provided by this study show high potential for selecting clams resistant to parasite load. Breeding for resistance may help limit the negative effects of climate change on clam production, as the prevalence of the parasite is predicted to increase under a future scenario of higher temperatures. Finally, the limited genetic correlation between resistance and growth suggests that breeding programs could incorporate dual selection without negative interactions.

## Introduction

Infectious diseases continue to be a major limit in fish and shellfish aquaculture, with production losses estimated to cost over 6 billion USD per annum, and far more in treatments such as vaccinations, cleaning, and antibiotics (1, 2). The frequency and severity of disease outbreaks are worsened by farming practices, unmonitored transfer of stocks for culture, and consequences of climate change such as rising sea-water temperature (3, 4). The latter has already been tied to an increase in host stress levels and pathogen virulence, as well as to changes in the geographical distribution of both pathogens and their hosts (5). Given the inherent difficulties in eliminating pathogens in the open-water environments most often used in aquaculture, genetic selection provides a feasible and sustainable solution to mitigating the impact of infectious diseases by identifying genetic factors associated with traits of interest such as disease resistance and growth. To date, genetic selection for the improvement of farmed aquatic animals remains only partly exploited (6), but the aquaculture sector has enormous potential and has already seen the development of tools with numerous applications at the commercial scale, mostly in fish and shrimp species (7, 8).

Over the past 20 years, a number of commercial and experimental breeding programs have been introduced for mollusks (9–18), many of which have obtained high genetic gains due to a combination of high heritability for economically favorable traits, high fecundity allowing for high selection pressure, and relatively short generation intervals (1–4 years for most species) (19–22). Recent estimates have shown that resistance to disease could be improved by as much as 15% per generation in mollusks by implementing individual or family-based selection (9). Despite the promising results for genetic gain in fish and shellfish species, it has been estimated that <10% of aquaculture production is based on genetically improved stocks, and most programs for bivalves are still experimental and small-scale, with few family lines and limited numbers of parents within each line (23). One of the reasons genetic selection has lagged behind in shellfish aquaculture is that, in order to design effective selective breeding programs, it is essential to obtaining pedigree information and accurate phenotype records, two aspects that are rendered far more complicated in shellfish species due to the fact that juveniles are too small at hatching (a few micrograms) to be physically tagged (24). Thus, distinguishing between families would evidently require raising large numbers of them separately, a method that comes with major disadvantages including high costs, extensive infrastructure, and the risk of confounding factors such as “tank effect” and “family effect,” which can significantly decrease the accuracy of estimated breeding values (25). Despite the difficulties associated with obtaining parentage information for shellfish, several recent studies have successfully applied SNP panels for parentage assignment in order to estimate genetic parameters of traits of commercial interest in mollusks (26–29). That said, most of the studies carried out so far seeking to evaluate the potential for genetic gain in bivalves have focused on traits such as disease resistance, growth rate, meat yield, and visual aspects such as shape and shell color, and relatively few have tackled the estimation of genetic parameters across all traits simultaneously (30). Balancing genetic gain for disease resistance with genetic gain for production traits is an essential step in designing rational breeding programs in order to evaluate the potential for genetic improvement across traits and, more importantly, to avoid selecting for a trait that may negatively affect other traits of interest.

The Manila clam is a bivalve species of particular commercial interest as it represents 25% of global mollusk production worldwide, and although it was introduced to Europe in the early seventies, European production currently accounts for only about 2% of global production (31–33). To date, few studies on additive genetic effects used for genetic selection have been carried out in this species. Genetic selection studies on the Manila clam have focused on shell length (divergent selection of extreme phenotypes) (34) and shell color (also divergent selection) (35). Heritability for larval and juvenile growth has recently been estimated through separate family rearing, running the risk of potentially overestimating the heritability due to confounding tank and family effects (36). Heritable resistance to *Vibrio tapetis* and shell repairing has also been reported after three generations of mass selection by SATMAR hatchery (37). In another clam, *Meretrix petechialis*, previous publications have also reported heritability for growth (38) and resistance to *Vibrio* pathogens (39). In *M. petechialis*, SNP markers were reported to be associated with resistance against *V. harveyi* and *V. parahaemolyticus* (40), and different levels of heritability were estimated according to the model used (linear, sire-dam threshold, or animal threshold model) (13). However, there is no information on the heritable component of resistance to *Perkinsus olseni* in the Manila clam. In the Eastern oyster *Crassostrea virginica*, previous hypotheses of heritable resistance to another protozoan parasite of the same genus, *P. marinus*, were confirmed through natural infections and mass selection (41–44). As infection levels within selected lines did not differ from those in the susceptible line, the authors suggested the development of tolerance within certain lines (as opposed to resistance *per se*). In addition, an important genotype-by-environment (GxE) interaction appeared to have been induced by highly variable ranges of salinity and water temperature between sites.

The protozoan parasite *P. olseni* has been identified as a major pathogen of the Manila clam, responsible for mass mortality of up to 70% in experimental infections, and affects clam production worldwide, including in the clam production zones of northern Italy (45, 46). The parasite induces the formation of nodules within the clam's gills that gradually spread to other tissues. In advanced stages of the disease, *P. olseni* causes lesions throughout various tissues that negatively affect the respiration and other physiological processes of the clam, leading to a reduction in growth, reproductive capacity, and condition index (47, 48). While this pathogen is consistently present in clam production zones such as the Venice Lagoon in Italy, with disease prevalence regularly reaching 80–100% in clams, uninfected Manila clams are detected in exposed populations during routine monitoring carried out by the Italian health authority and research organization for animal health and food safety (IZSVe). Given the chronic nature of the disease, mortality is not always observed within the 2-year grow-out period typically seen in Italy, and populations frequently display a wide range of infection levels.

The two most important abiotic parameters associated with the infectivity of *P. olseni* are temperature and salinity (49, 50). These two factors are highly variable in normal lagoon environments, and these variations are already amplified by current climate change, causing important shifts in ecosystems and increased stress levels to aquatic organisms, especially at the local scale (51, 52). Given the metabolic effort necessary for marine organisms such as clams to sustain normal physiological functions in conditions at the far end of their thermal range, the consequent decline of their immune capacity, coupled with the increased virulence of pathogens such as *P. olseni* under the same conditions, can lead to devastating mortality events, which will undoubtedly lead to an increase of frequency under future climate scenarios (53). In light of the potential increase in parasite prevalence and the physiological stress induced by rising seawater temperatures, as well as the difficulties associated with treating infectious diseases in bivalve species, selection for resistance to *P. olseni* may represent a sustainable solution for clam aquaculture in the near future.

In this study, a 245-SNP panel for parentage assignment was developed and used to genotype individual Manila clam samples from a large-scale long-term field experiment with the protozoan parasite, *P. olseni*, in commercial aquaculture conditions. Finally, phenotypic, pedigree, and genomic information was integrated to estimate genetic parameters of the traits recorded in order to evaluate the feasibility of carrying out effective genetic selection for multiple production traits and lay the basis for designing balanced breeding programs in hatcheries.

## Materials and Methods

### Field Challenge Site and Biological Material

An experimental population of clams was produced by mass spawning in May 2016 at the French hatchery SATMAR (Barfleur, France) from 109 adult clams of hatchery broodstock, according to a two-factorial mating design: 31 dams × 25 sires (775 putative families) and 22 dams × 32 sires (704 putative families), representing potentially 1,479 F1 families. Soon after fertilization, the larvae from the two pools were mixed together and reared at the SATMAR hatchery in a common tank in order to avoid confounding between genetic and common environment effects. After spawning, the parents were sacrificed and a piece of mantle from each of the 109 parent clams was kept in 99% ethanol for future DNA extraction and genotyping. In September 2017 a batch of 10,000 progenies was seeded in Chioggia at 1.1 g (see section Monthly Monitoring of Field Challenge), a commercial grow-out site in the Venice lagoon, Italy, where there is a high prevalence of the pathogen *P. olseni* [87% in 2016, Regional epidemiological annual report, by the National Reference Center for Fish, Mollusc and Crustacean Diseases (IZSVe), Legnaro (PD)].

### Monthly Monitoring

**Figure 1:**
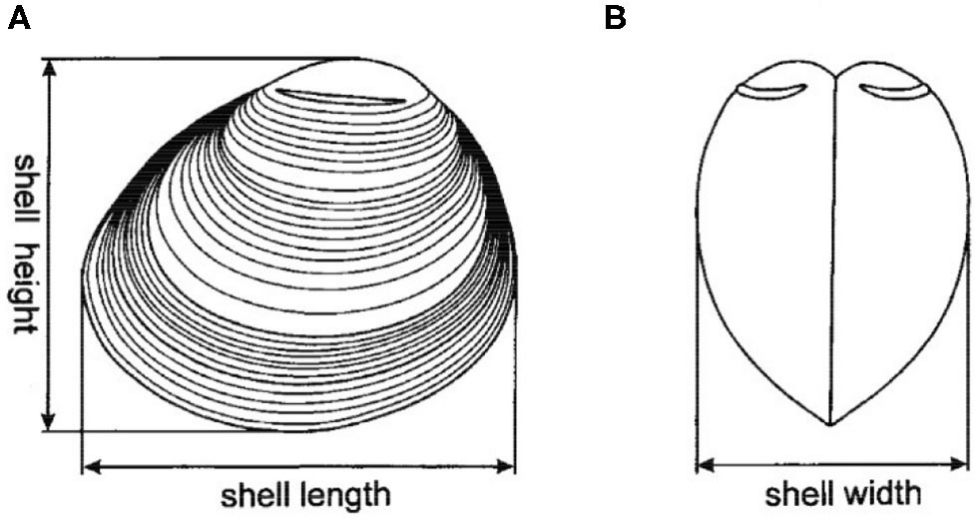
Shell dimensions measured for all F1 clams from both Chioggia and Marennes clam groups, adapted from Grigorovich et al. (54). **(A)** Lateral view; **(B)** Frontal view.

Over the course of 1 year, the prevalence and intensity of *P. olseni* in the gill tissues of the F1 clams was evaluated monthly since the time of seeding. Each month, about 30 clams were sampled for whole weight, shell weight, and shell length, and the gills were dissected and frozen in liquid nitrogen for subsequent DNA extraction. Biometric measurements allowed us to follow growth parameters of the clam experimental population [Figure 1, (54)]. Disease intensity was measured based on quantification of parasite DNA in the total DNA from the gill sample (protocol described below), and prevalence was considered as the percentage of clams in which parasite DNA was detected (>LOD), though not necessarily quantifiable. This monthly sampling strategy was employed in order to monitor disease progression within the F1 clams, bearing in mind the annual variability of disease intensity and the historical risk of mass mortality in the area.

### Large-Scale Phenotype Recording and DNA Sampling

Mass sampling was carried out in September 2018, when 50% of the F1 clams were positive (>LOD) to *P. olseni* infestation and, having spent 1 year in grow-out conditions, had reached commercial size. This threshold of infestation was chosen according to Chapuis et al., allowing better settings with which to maximize differences between families and thus increase the precision of the genetic parameter estimates (55). The number of samples was set at *n* > 1,000 in order to expect a mean of 18–20 progenies per parent, for a relevant estimation of genetic parameters in a mixed family design that accounts for a variable number of sibs per parent (56). This is based on the estimations described by Dupont-Nivet et al. (57) demonstrating effective population size (Ne) and genetic variability to be maintained in a population size of *n* = 1,000 for full factorial mating designs considering 50 × 50 parents.

In our study, 1,000 clams were individually weighed whole, then, meat and shell were separated and the shell was weighed after draining. Sex was determined, where possible, by visualization under a microscope of a gonad smear. Both gills were collected from each individual clam and stored in 1.5 ml 70% ethanol. Gill tissue was selected for the DNA extraction as it is the first tissue to become infected with *P. olseni*, and thus it was possible to carry out total DNA extraction from the same sample for both parasite quantification and host genotyping (see below: *P. olseni* quantification). All of the individual phenotype data was recorded in the INFAQUA database system dedicated to data storage and provided by SYSAAF. A description of all the traits measured can be found in Table 1.

**Table 1:** Description of the traits recorded for each individual clam during the large-scale phenotyping and DNA sampling.

**Trait**	**Unit**	**Description**
Total weight	g	Whole individual weight
Shell length	mm	Maximum length between the posterior and anterior parts (see Figure 1)
Shell weight	g	Weight of cleaned shell, without meat
Parasite load	Copies/uL	Number of copies of *Perkinsus olseni* DNA in 1ul of total extracted DNA (concentrations normalized at 10ng/ul of total DNA)

*All measurements (except parasite load, which was acquired after DNA extraction) were recorded directly in the INFAQUA database*.

### DNA Extractions and *P. olseni* Quantification

Individual total DNA extraction was carried out from gill tissue samples by using Invisorb® DNA Tissue HTS 96 Kit according to the manufacturer's instructions. Then, *P. olseni* load in individual clams was estimated by real-time PCR using specific primers for *P. olseni* to amplify 1μl total DNA extracted from one whole clam gill (58). Briefly, samples were homogenized with a benchtop TissueLyser® in 80 μl PBS buffer with silica beads. Lysis with proteinase K was carried out overnight at 56°C prior to isopropanol precipitation, and DNA was eluted in 100 μl MilliQ water. Total DNA concentration was measured by spectrophotometry (Nanodrop®) and quality of the DNA was assessed by electrophoresis on 1% agarose gel. Total DNA samples were normalized at 10 ng.μl^−1^ and PCR assays were performed in a total volume of 25 μl containing 1 μl genomic DNA, 12.5 μl Sybergreen PCR Master mix, 10.5 μl clean H_2_O, and 0.5 μl both forward and reverse primers (10 μM). PCR cycles were performed using a 7,300 Real Time PCR system from Applied Biosystems as follows: 95°C for 10 min; 40 cycles at 95°C for 30 s and 60°C for 1 min followed by 72°C for 5 min and a melting curve at 95°C for 15 s; 60°C for 30 s and 95°C for 15 s (58). Three separate levels of infestation could then be determined: “negative” (=0), “positive” (>LOD), and “quantifiable” (>LOQ).

### Development of SNP Panel for Parentage Assignment

In order to determine pedigree, a panel of SNPs was developed based on pooled DNA sequencing using the genetic material of the 109 parents bred to create the F1 cohort.

#### PoolSeq of Parental DNA From Venice Lagoon Population and VIVALDI Experimental Population

Fifty individuals of *R. philippinarum* were collected in 2011 from a clam production site in the Venice lagoon, and individual samples of mantle tissue (stored in ethanol) of the 109 Manila clams used as broodstock by the SATMAR hatchery were sent to the BCA laboratory for DNA extraction in March 2018.

Extractions were carried out individually using Invisorb® DNA Tissue Kit (STRATEC Biomedical). Total DNA was quantified by Qubit® 2.0 fluorometer and quality was evaluated on a 1% agarose gel. For the Venice lagoon samples, two equimolar pools of 25 individuals/pool were prepared, and the DNA was fragmented using Covaris and prepared for sequencing in 2017. For the broodstock individuals, samples were allocated to equimolar “lower quality” (LQ) and “higher quality” (HQ) pools based on molecular weight as visualized by electrophoresis.

Libraries for each pool (2 × Venice lagoon clams, and 2 × broodstock) were prepared in triplicate using TruSeq® DNA Library Prep (Illumina). After Qubit 2.0 quantification and quality evaluation using Agilent 2100 Bioanalyzer, the triplicate libraries were pooled (equimolar) and sent for Illumina HiSeq 4000 for 150-bp paired-end sequencing at the Genome Center of the University of California—Davis. Reads were trimmed to remove low-quality reads using TRIMMOMATIC, and CLC Genomics Workbench was used for aligning raw reads to the *R. philippinarum* draft reference genome (59). SNP identification was carried out using PoPoolation2, a pipeline for analyzing pooled next generation sequencing data, which builds on open source tools (33).

#### SNP Filtering by Selection Criteria

SNPs detected by the PoPoolation pipeline in both Venice lagoon clams and hatchery broodstock DNA were selected based on the criteria listed in Table 2.

**Table 2:** Criteria for SNP filtering to select candidate SNPs.

**Criteria**	**Explanation**
Minor allele frequency	Only SNPs with a minor allele frequency greater than or equal to 0.3 were considered
Minimum and maximum coverage	In order to avoid selecting SNPs present in long tandem-repeat regions, candidate markers were selected within genomic regions showing a sequencing coverage ranging between 25× and 100×
Minimum stable flanking region	In order to design specific primers to amplify each genetic marker on the chip, each SNP must have a non-variable up- and down-stream flanking region of at least 100 bp
Presence in both hatchery broodstock and Venice lagoon populations	This criterion was chosen in order to provide a panel that could assign parentage not only for the cohort produced from the hatchery population, but also more broadly in other Manila clam populations
Number of alternative alleles	Considering that diallelic SNPs are easier to design probes for, multiallelic markers were not considered
Contig proximity	In order to decrease the risk of using linked genetic markers, no more than one SNP per contig of the *R. philippinarum* reference genome was selected
Mirror SNPs	Mirror SNPs are those targeting complementary bases (i.e., A/T or C/G) for which single probes present the same fluorescence, hence they require a secondary probe to distinguish between them. These complementary base SNPs were removed from the list to avoid requiring secondary probes and introducing additional signal analysis complexity

#### SNP-Chip Design

The 100 bp flanking sequences for the SNPs that matched all of the selection criteria were shared with Labogena Genotyping Company, which designed probe sequences using Illumina DesignStudio® Sequencing Assay Designer, following supplier recommendations. From the initial 250 probes designed, single probes for 245 SNPs were placed on an Illumina Infinium® genotyping chip able to genotype 96 samples/chip.

### Genotyping and Parentage Assignment

DNA samples were genotyped (4 ul of genomic DNA with a minimum concentration 10 ng.ul^−1^) using the Infinium chip and analyzed on a fully automated Illumina iScan® platform, following supplier recommendations. Genotype quality control was carried out using GenomeStudio® software, according to the following standard Labogena clustering procedure: run of Illumina automated clustering algorithm (34): call rate calculation for every sample; Exclusion of outlier samples; Second run of Illumina automated clustering algorithm; Readjustment of clustering on every SNP, according to Labogena internal protocol; Exclusion of SNPs presenting ambiguous graphical cluster profiles; Second evaluation of call rate per sample, using last clustering; Exclusion of samples with call rates under NorM R threshold value of 0.2.

Genotypes that met the above clustering criteria were analyzed using AccurAssign® software from Labogena, which carries out parentage assignment based on both maximum likelihood and exclusion methods, using the following parameters: 1% of genotyping errors, minimum of 70% of positive markers for each sample, maximum of 3 mismatches between progeny and parent couple. Furthermore, the mating plan used to produce the populations tested (7 hatchery families and 109 hatchery parents including 96 F1 descendants) was included in the software parameters in order to correct assignments lying outside of the mating plan (35).

### Genetic Variability

Estimations of effective population size (*N*_*e*_) were used to estimate the number of parents necessary to maintain genetic diversity, or allele frequencies, within a population. *N*_*e*_ can be calculated assuming random mating and equal parent representation among the F1 population, according to Kimura and Crow (60):

$$\begin{array}{l} N_{e}=\frac{4}{\frac{1}{N_{em}}+\frac{1}{N_{ef}}} \end{array}$$

where *N*_*em*_ and *N*_*ef*_ are the number of effective male (sire) and female (dam) parents, respectively.

Equal family representation is rarely observed in mass spawning of marine fish and shellfish (25), thus there is often a tendency toward strong overrepresentation in the F1 generation of only a few of the parents participating to the spawning. This means a high risk of inbreeding and loss of genetic diversity. Estimated *N*_*e*_ can give an indication of the risk of inbreeding, and in order to increase the accuracy of inbreeding estimations, the variability of family representation can be considered when calculating Ne, according to Harney et al. (29) and Chevassus (61), expressed as:

$$\begin{array}{l} \frac{4}{N_{e}}=\frac{\left(K_{m}+\frac{V_{m}}{K_{m}}\right)+\left(K_{f}+\frac{V_{f}}{K_{f}}\right)-2}{N-2} \end{array}$$

where *N* is the total number of individuals, *K*_*m*_ and *K*_*f*_ are the average number of progeny per sire and dam, and *V*_*m*_ and *V*_*f*_ are the variance of the number of progeny between sires and dams.

The rate of inbreeding per generation (*F*_*t*_) can be calculated for a population and a number of generations (*t*) based on the *N*_*e*_, according to Falconer (62), expressed as:

$$\begin{array}{l} F_{t}=1-{\left[1-\frac{1}{2N_{e}}\right]}^{t} \end{array}$$

### Estimation of Genetic Parameters

Data were checked before analyses to eliminate outliers: measurements that were less than or greater than the mean + 4 standard deviations were systematically eliminated. Genetic parameters were estimated using two programs derived from the software BLUPF90: GIBBSF90 for continuous traits and the THRGIBBSF90 threshold model in cases where at least one non-continuous trait (in our study this pertains to resistance to *P. olseni*) was included in the model. Genetic parameters were estimated using pedigree information with the following mixed animal model (63, 64):

$$\begin{array}{l} y=X\beta +Za+\varepsilon \end{array}$$

where ***y*** is a vector of *n* observations on *p* traits, *p* = 1 in the case of univariate analysis for the estimation of heritability, and *p* = 2 in the case of bivariate analysis for the estimation of genetic correlations. **β** is a vector of fixed effect, we consider only one fixed effect for the batch (Chioggia). **a** is a vector of additive animal genetic effects distributed following a normal distribution N$\left(0,\;\text{A}\sigma_{a}^{2}\right)$ where **A** is the pedigree relationship matrix and $\sigma_{a}^{2}$ the (*p* × *p*) genetic additive variance-covariance matrix. *X* and *Z* are incidence matrices for fixed and genetic additive effects, respectively, and **ε** is the residual component distributed following a normal distribution N$\left(0,\;I\sigma_{e}^{2}\right)$, where ***I*** is the identity matrix and $\sigma_{e}^{2}$ the (*p* × *p*) residual variance-covariance matrix.

Heritability (*h*^2^) was calculated according the following formula:

$$\begin{array}{l} h^{2}=\frac{{\sigma^{2}}_{a}}{{\sigma^{2}}_{a}+\;{\sigma^{2}}_{e}} \end{array}$$

where $\sigma_{a}^{2}$ is the genetic additive variance related to pedigree and residual variance $\sigma_{e}^{2}$. In the case of bivariate analyses (i.e., *p* = 1), genetic additive covariance was estimated and genetic correlations (*R*_*g*_) between two traits were calculated as follows:

$$\begin{array}{l} {R_{g}}_{\left(trait1;\;trait2\right)}=\frac{{Cov_{a}}_{\left(trait1;\;trait2\right)}}{{\sigma_{a}}_{\left(trait1\right)}\times {\sigma_{a}}_{\left(trait2\right)}} \end{array}$$

where *Co*_*v*_*a*_(*trait*1; *trait*2)_ is the genetic additive covariance between trait 1 and trait 2, _σ_*a*_(*trait*1)_ and _σ_*a*_(*trait*2)_ are the standard deviations of additive genetic effect for trait 1 and trait 2, respectively.

## Results

### Monthly Monitoring of Field Challenge

Sampling occurred monthly over the course of 1 year, from the time of seeding (T0) to the final sampling event. Time-points and days post-seeding (dps) are detailed in Supplementary Material.

**Figure 2:**
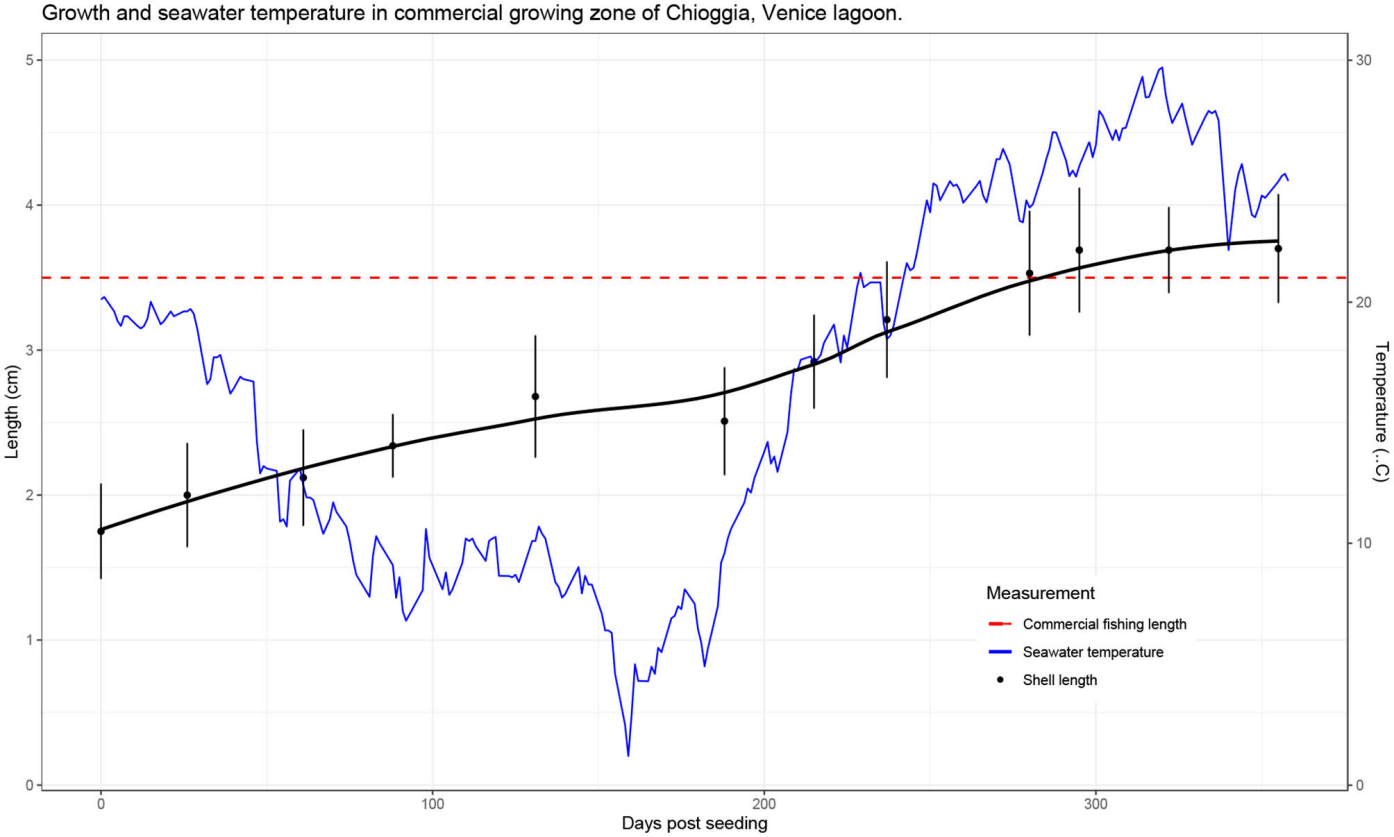
Clam growth (shell length) as recorded from September 2017 to August 2018 (355 days total). Shell length is the mean value measured at each time point, and error bars represent standard deviations. Temperature measurements were recorded daily by the Hydrobiological Station of Chioggia at the entrance of the lagoon (records available at: https://chioggia.biologia.unipd.it/en/the-database/parameters-of-lagoon/).

At the time of seeding in the grow-out area, average shell length was 1.75 cm (SD = 0.33) and average whole weight was 1.115 g (SD = 0.626). At the final sampling date, 355 days post seeding (dps), average shell length was 3.48 cm (SD = 0.37) and average whole weight was 11.918 g (SD = 3.577). Growth, represented as average shell length over time, is reported in Figure 2.

At each time-point over the course of the year-long monitoring, gill samples were collected from each of the clams and used for DNA extraction. Real-time PCR was carried out at the IZSve to quantify the parasite DNA for all samples from T4 (130 dps) to the final sampling (355 dps). Diagnosis was not carried out on samples from T0 to T3 (0 to 93 dps) as parasite levels were below the limit of detection (LOD). Prevalence (considered as the percent of samples above LOD) began to increase at T5 (187 dps), where 4.5% of the samples tested positive for *P. olseni* (>10 copies.ng^−1^). Prevalence continued to increase, reaching 36.7% at T10 (322 dps), 1 month prior to the final sampling event (355 dps). The rise in *P. olseni* prevalence follows a trend similar to that of the average monthly water temperature, as it is well-known that the parasite is more virulent at higher temperatures. Final sampling was carried out when clams reached the commercial size for harvesting and at the end of the warmest summer months, when it was expected the prevalence would rise considerably. After diagnosis of all the sampled F1 individuals, the final prevalence was 51.5%.

### Large-Scale Phenotyping

During the mass sampling event in Chioggia, clams were measured for total weight, shell weight, and shell length. Sex was recorded whenever it was possible to unambiguously assess gonadal sex, and final male to female sex ratio was 47:53 (Supplementary Material). Parasite load was measured in *Perkinsus*-positive individuals above the limit of quantification (Table 3). Positive individuals (above the limit of detection) were scored as positive, but not quantifiable. All remaining individuals were considered *Perkinsus*-free (negative).

**Table 3:** Full summary of traits recorded in Chioggia F1 group.

**Chioggia**	***n***	**Mean ± SD**	**Min**	**Max**
Total weight TW (g)	1,087	11.89 ± 3.63	2.8	26.9
Shell length SL (mm)	1,088	36.94 ± 3.78	23.18	50.55
Shell weight SW (g)	1,058	6.725 ± 2.09	1.86	15.21
Log10 P. olseni^*^ (copies.μl^−1^)	544	3.2 ± 0.67	2.01	5.23

* *Descriptive statistics for this trait are shown only for the 544 samples that contained quantifiable amounts of P. olseni DNA (>LOQ)*.

Real-time PCR on DNA extracted from clam gills revealed that 244 clams were negative for *P. olseni* DNA, 268 showed detectable parasite DNA, but below the limit of quantification (LOQ: > 30 threshold cycle (Ct); equivalent to 100 copies of *P. olseni* DNA), and 544 samples contained a quantifiable amount of *P. olseni* DNA (Table 4).

**Table 4:** Means and SD for shell length (SL), shell weight (SW), and total weight (TW), presented in three clam groups depending on the parasite load (negative; below LOQ; quantifiable for parasite DNA) for experimental F1 clams in the Chioggia field challenge.

**Parasite load class**	***n***	**Copies.ul^**−1**^**	**SL**	**SW**	**TW**
Quantifiable	544	5265 ± 1189	36,2 ± 3,88	6,33 ± 2,09	11,2 ± 3,60
<LOQ	268	NA	37,3 ± 3,70	7,10 ± 2,10	12,5 ± 3,54
NEG	244	NA	38,1 ± 3,05	7,18 ± 1,85	12,7 ± 3,21

### Parentage Assignment

Of the 1,056 samples analyzed for parasite load, 992 samples had sufficient remaining biological material for genotyping, and 601 samples were successfully genotyped with the SNP-chip (61% success rate). Parentage assignment to a single parent pair was successful for 246 (41%) of the genotyped samples.

### Genetic Variability and Estimation of Genetic Parameters

Of the 56 sires and 53 dams used to produce the F1 clams, parentage assignment showed that most were represented in the F1 clams (Table 5). Despite the limited success of assignments (*n* = 246), 163 families (or parent-pairs) containing 42 sires and 45 dams were observed out of the 1,479 putative full-sib families from the two-factorial mating design.

**Table 5:** Genetic variability: parent representation and effective size (*N*_*e*_).

	**Chioggia**
Assigned offspring	246
Expected sires	56
Expected dams	53
Expected families	1,479
Observed sires	42
Observed dams	45
Observed families	163
Expected N_*e*_	108.9
Observed N_*e*_	86.9
Observed N_*e*_ with offspring variance	39.0

The initial expected effective population size (*N*_*e*_), based on number of parents that were used in the mass spawning, was 108.9. After parentage assignment of the F1 clams, the observed *N*_*e*_ (calculated based on the number of parents that were actually represented in the F1 groups) was 86.9. When considering the variability in representation of the parents, i.e. the variance of the number of offspring per sire and dam, (Table 5; “Observed Ne with offspring variance”), the *N*_*e*_ drops down to 39.0. The inbreeding rate per generation based on the observed *N*_*e*_ is 0.58%, while the inbreeding rate considering N_e_ with offspring variance (i.e., disregarding pedigree information) is 1.28%.

Heritability (*h*^2^) was estimated for each trait, and genetic correlations (Rg_trait_) between traits were calculated pairwise (Table 6). Individually estimated heritability estimates for biometric traits range from 0.23 ± 0.11 (total weight) to 0.35 ± 0.13 (shell weight). Heritability for parasite load (0.51 ± 0.20) is high. The high error (standard deviation) observed can likely be attributed to the low number of assigned individuals.

**Table 6:** Estimated heritability (*h*^2^) of each trait (total weight [TW], shell weight [SW], shell length [SL], and parasite load [PL]) in bold along the diagonal, and genetic correlations (R_*g*_) between traits above the diagonal, with standard deviations in brackets.

	***h*^2^ and *R*_*g* t*rait*_ for traits measured in F1 clams raised for 1 year in commercial conditions**			
	TW	SL	SW	PL
TW	**0.23 [0.11]**	0.9 [0.07]	0.97 [0.03]	−0.16 [0.37]
SL		**0.29 [0.13]**	0.78 [0.13]	0.18 [0.37]
SW			**0.35 [0.13]**	−0.21 [0.33]
PL				**0.51 [0.20]**

Genetic correlations between biometric traits were high, ranging between 0.78 ± 0.13 (SL vs. SW) and 0.97 ± 0.03 (SW vs. TW). The average measured values for biometric traits between negative, detectably (<LOQ), and quantifiably infected clams (Table 4) tended to decrease with increasing parasite quantity. However, the seemingly negative genetic correlations between parasite load and biometric traits were not significant due to high standard error rates (Table 6).

## Discussion

Shifts in global temperature levels associated with climate change and other anthropogenic pressures are having increasingly negative impacts on marine ecosystems, namely with regard to pathogens. The geographical ranges of hosts and pathogens are being modified and their respective resistance capacity and virulence altered, leading to devastating mortality events due to both emerging and previously described pathogens. The viability of bivalve production will largely depend on the ability of the species to adapt to the future environmental conditions, through either natural or artificial selection of those individuals that are genetically more resistant to pathogens and stressors associated with global warming (65). To assess the potential of breeding programs to mitigate the effects of climate change, however, it is necessary to precisely estimate the additive genetic variance underlying the response of bivalves to infectious diseases associated with global warming. To this end, rigorous experiments are required where a large number of families are challenged under controlled conditions. Equally important is to try and simulate as much as possible the “normal” conditions that are experienced by farmed animals for commercial production. This is particularly relevant in bivalves as it has been already reported that the infectious diseases are often caused by a combination of abiotic and biotic factors, which include water temperature, specific pathogens, and alterations of the microbial communities associated to the host (66). Finally, it is most important to evaluate whether resistance to climate-change associated diseases is genetically correlated with other traits, which are of potential interest for producers, such as size and shape. In fact, breeding for increased growth in addition to disease resistance will boost the efficiency and quality of bivalve production, a key target for modernization of the sector (4).

**Figure 3:**
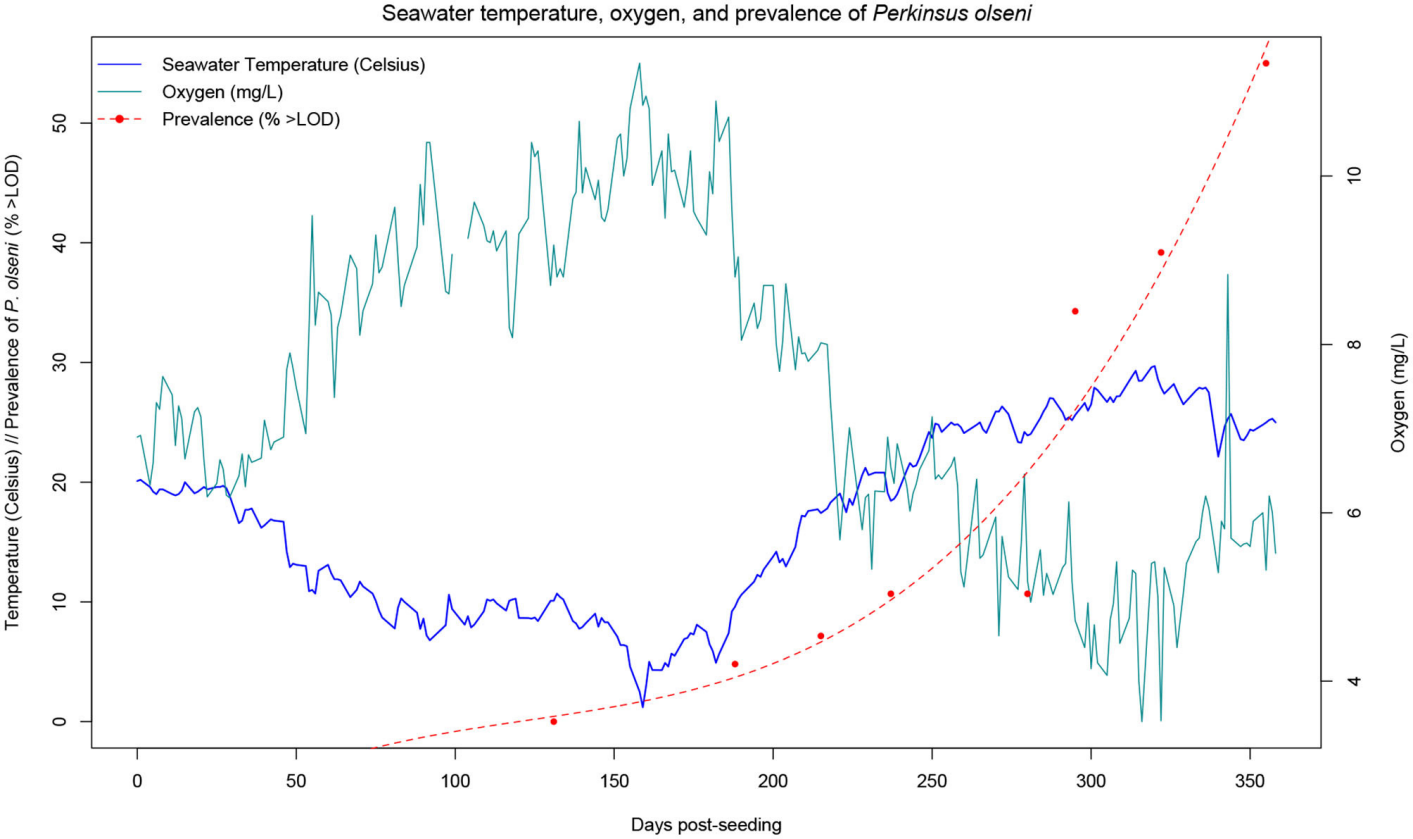
Monthly average seawater temperature in Chioggia, Venice lagoon, and prevalence of P. olseni in clams. Prevalence is considered at the percentage of samples for which the parasite DNA was detected (but not necessarily quantifiable). Temperature and dissolved oxygen measurements were recorded daily by the Hydrobiological Station of Chioggia at the entrance of the lagoon (records available at: https://chioggia.biologia.unipd.it/en/the-database/parameters-of-lagoon/).

In this paper, we demonstrate that it is possible to carry out a long-term field challenge in the Manila clam, with the protozoan parasite *P. olseni* under commercial conditions. Analysis of the growth trajectory confirmed previous findings for on-growing sites in Adriatic Sea lagoons, including the Venice lagoon (49), with continuous increase in length with the exception of winter due to the lower temperatures and phytoplankton availability. Numerous studies have recorded growth parameters of the Manila clam in various environments, aiming to assess economic and biologic productivity, and evaluating the benefit of introducing the species for aquaculture. The Venice lagoon provides a highly favorable environment for clam growth, in that commercial size (35 mm) can be reached in <2 years, compared to 3 years in less favorable production environments (67–69). In our study, clams were seeded at a mean shell length of 17.5 mm and reached commercial size of >35 mm within 1 year (Figure 2). While higher temperatures are associated with higher growth rates, they are also accompanied by a higher prevalence of infections from thermophile pathogens. During our study, *P. olseni* was first detected in 4.5% of the F1 clams in March (T5), increasing steadily with the seasonal rise in seawater temperature. Optimal temperatures for proliferation of *P. olseni* have been shown to range between 20°C and 34°C (50), therefore it is not surprising that in summer months, we observed that disease prevalence showed an exponential growth reaching a value higher than 50% (Figure 3). In the Venice lagoon, seawater temperatures in summer often reach over 28°C, while available oxygen rapidly drops in the water column. Clams can survive anoxic conditions for at least 12 h, such as during transport or in tidal zones. However, prolonged exposure to high seawater temperatures and associated low oxygen levels are likely to be one of the key factors underlying the rise in prevalence and infection intensity levels of *P. olseni* observed in our study as well as in numerous other studies (50, 70–72). Low water temperature (<13°C) and low salinity (<15 psu) have previously been suggested to suppress *in-vivo* propagation of the pathogen in the Manila clam (73). In the related species *P. marinus*, a well-known oyster pathogen, trophozoites, the vegetative multiplication phase that occurs in host tissues, were shown to have the highest propagation rate *in vitro* at 28°C, indicating that high temperatures inhibit the host immune functions as well as increase the ability of the pathogen to spread within the host (50). Given the predicted rise in global seawater temperatures, it is likely that bivalve mollusks will be pushed further to the limits of their current tolerance ranges, leaving them fewer resources with which to combat infections, while at the same time these same conditions will be far more favorable to pathogens such as *P. olseni*. While this microparasite normally induces only sporadic mortality, albeit affecting host performance, under extreme environmental conditions it could provoke massive mortalities such as those reported in 2014 in the Venice lagoon (34). The observed pattern of constant growth and disease prevalence increasing exponentially only in the late summer also suggests that selective breeding for faster growing animals might confer further protection against for *P. olseni*, in addition to direct genetic selection for disease resistance. Fast growth could allow for collection of commercial sized clams earlier on, thereby decreasing the risk of sudden mortalities induced by hypoxic, high temperature conditions in the summer months.

As mentioned previously, an important element for efficient implementation of selective breeding is reliable estimation of genetic parameters for selected traits. Likewise, these parameters represent crucial information for minimize inbreeding. In both cases, knowledge of the selection candidate pedigree is key. This is generally achieved by either keeping the different families separated, physically tagging individuals, reconstructing *a posteriori* parentage by means of molecular or genetic markers, or by using a combination of these three approaches. In the case of aquaculture species, parentage assignment based on DNA fingerprinting is a useful tool as it does not require investment in large and costly facilities in which to rear separate families. As no such tool was available for the Manila clam, we adopted a whole-genome pool sequencing approach to identify a large set of SNPs from two clam population samples, which included the F1 sires and dams. A similar strategy for SNP discovery was implemented in other bivalves such as European and Pacific oyster (74). From the large SNP set obtained, a 245 SNP panel was selected and used for the development of a SNP array. A first validation of this SNP array on known families indicated that it was able to assign up to 81.2% (data not shown). Although not ideal, the obtained conversion rate appeared satisfactory considering the well-known problems in SNP genotyping in bivalves, largely due to the extremely high polymorphism (e.g., one SNP every 15 bp), and the assignment rate is comparable to the values reported for other fish and shellfish species, such as the Pacific oyster, *C. gigas*, in which assignment rates in recent studies vary between 72 and 94% (75). The rate of successful parentage assignment on field samples (41%) was considerably less satisfactory. It seems partially explained by the low positive genotype rate (61%). It is not clear why field samples showed much lower genotyping rate. Quality tests were carried out to determine the best tissue for DNA extraction, and results indicated that gill tissue yielded sufficient quality and quantity (see Supplementary Material). Several factors might account for the reduction in parentage assignment rate. First, a large number of sires and dams was used to produce the F1 population, making unambiguous assignment to a single parent pair more difficult, although generally 100 SNPs are sufficient for reliable assignment. While the initial validation trial with known families was promising, we cannot exclude that the quality of the SNPs selected for the array was insufficient for large-scale genotyping and assignment due to the occurrence of null alleles, duplicated genomic regions, and transposable elements, all of which are documented as being frequent issues in molluscan genomes (20, 24). The possibility of these factors affecting SNP quality in the array is exacerbated by the fact that the draft genome used for marker selection was not annotated, thus markers could not be selected in more stable coding regions. Finally, the experimental on-growing area was preventively vacated using a mechanical fishing dredger and protected with special nets, though it cannot be excluded that a small percentage of missing assignments could be associated to unrelated clams having settled in the experimental area. Overall, parentage assignment in the Manila clam certainly has significant room for improvement, and estimates for genetic parameters must be considered preliminary and interpreted with caution. However, despite the less than optimal efficiency, the number of reconstructed families allowed the estimation of population structure and genetic parameters for all the measured traits.

Based on the pedigree of the 246 assigned F1 individuals, 75% of sires and 85% of dams (of the 109 parents used in total) appeared to have contributed to the offspring at the time of analysis, over 2 years after the reproductive event. Comparable values of parent representation were reported in four hatchery populations of Pacific oyster, ranging between 64 and 100% for sires and 68–96% for dams. The clam progeny used here were obtained using mass spawning, a technique where all sires and dams are mixed together and release sperms and eggs in the same tank. In aquatic species such as bivalves, where a large number of animals participate in the spawning event, often only a limited number of sires and dams significantly contribute to the offspring, strongly reducing the effective population size (76, 77). The relatively unbiased contribution from a large percentage of parents observed here might allow for successfully obtaining offspring that are sufficiently diverse at the genetic level, without resorting to more troublesome practices such as controlled one-to-one crosses. The observed population size (*N*_*e*_ = 86.9), excluding reproductive success variance, was close to the expected *N*_*e*_ of 108.9), indicating that high genetic variability may be conserved when using DNA-parentage assignment to manage broodstock and limit over-representation of certain parents. When considering variance in reproductive success (i.e., excluding pedigree information to manage inbreeding) *N*_*e*_ dropped to 39.0. When the observed *N*_*e*_ values were used to estimate inbreeding rate after one generation (Δ*F* = 1/(2^*^N_e_), estimated Δ*F* was lower (0.006) or slightly higher (0.013) than the internationally recommend threshold (Δ*F* < 0.01) to avoid excessive increase of inbreeding rate in farmed animals (78). In other aquatic species such as rainbow trout, Δ*F* ranges between 1 and 2.2% per generation (79), while in others is significantly higher as in the case of the banana shrimp, which shows an average inbreeding rate of about 4% per generation (80). Even considering the higher Δ*F* value estimated in clams, mass spawning might allow breeding programs to remain within the internationally proposed maximums suggested by the FAO for fish breeders in order to assure long term management of genetic variability (81, 82). Maintaining sufficient genetic variability is crucial in breeding programs, especially in the current context of climate change, which already exerts high selective pressure on marine species by pushing them to the edges of their tolerance ranges for numerous abiotic factors (83).

Regarding effective population size, genetic parameters of all recorded traits could be estimated despite the limited efficiency in parentage assignment. Heritability estimates for total weight, shell length and shell weight are intermediate (0.23–0.35). These heritability estimates fall within the range of realized heritability previously reported by mass selection for shell length of *h*^2^ = 0.26 in the Manila clam (34). While these estimates have non-negligible error rates, likely due to the limited number of individuals assigned, they are the first reported by using a BLUP animal model in this species.

Total weight is highly correlated with shell weight (0.97) and shell length (0.9). This indicates that rapid assessment of growth can be done on the field by selecting only shell length. Similarly, as shell weight is highly correlated to total weight, it means that measurement of growth on sibs could be delayed and assessed by considering shell weight. However, as shell weight and length are less genetically correlated (0.78), and genetic correlation is lower than 0.8, these two last traits have to be considered as separate traits.

When considering genetic correlations between traits, the correlated response to selection of one trait on another trait highlights the possible benefit the 52% higher heritability of SW compared to TW (0.35 vs. 0.23). Based on the Falconer (1960) formula (62) to estimate correlated response to selection, applying selection on SW in order to improve TW would be 19.7% more efficient than direct selection of TW at the same selection pressure (see Supplementary Material). The higher heritability of SW compared to TW is likely due to higher environmental variance for TW, though the high correlation between the two traits (0.97) indicates that family ranking will remain the same through indirect selection. That said, direct selection of candidates based on their shell weight is not possible. But if this information were available on sibs or for future genomic approaches, it should be recommended to consider SW rather than TW, as error variance increases in the latter due to variable water presence in the animal.

Regarding disease resistance, heritability for parasite load with *P. olseni* was estimated at 0.51 ± 0.20, indicating high potential for genetic improvement of this trait, though results should be interpreted with caution due to the low number of assigned individuals. Infections due to *Perkinsus*, with time and given the right environmental conditions, are likely to affect up to 100% of a Manila clam population. In the present study parasite load was recorded after 1 year on the field. In the past, natural spat was routinely used to seed on-growing areas, therefore clams were exposed to potential infection with *P. olseni* for a longer period (1.5–2 years) as the entire life-cycle occurred in the field. In recent years a dramatic drop in natural recruitment has forced producers to use hatchery-produced spat of larger size (12–15 mm), with a reduced potential exposure to *P. olseni*, much more closely resembling the experimental protocol that was followed in this study. It is important to consider that individual parasite load 1 year post-seeding was used a as a measure of resistance, which, in the case of chronic infections, may be an indirect assessment for resistance. In *Penaeus monodon* a limited genetic correlation was reported between gill-associated virus (GAV) load and survival (84). Moreover, and as reported in *C. virginica* regarding infection with *P. marinus* (see Introduction), evaluations for resistance to disease should integrate potential GxE effects brought on by variations in salinity or temperature between sites. Our results would thus greatly benefit from validation through future evaluations of response to realized selection experiments and larger data sets.

Interestingly, there was no genetic correlation between parasite load and growth-associated traits. Because of the limited number of analyzed individuals, however, the standard error for these estimates is large and caution should be exerted when considering genetic correlation. If the evidence of no genetic correlation between growth and parasite load will be confirmed using a large set of animals, then selection for one of these two traits may have no direct effect on the performance of the other, indicating that dual genetic selection could be implemented without running the risk of inducing a negative effect.

Understanding the genetic correlations between various phenotypes is essential, as focusing selection on certain traits of interest can provoke devastating effects on other health-related traits, as demonstrated extensively in terrestrial livestock selection (85, 86). Overall, the high heritability estimates for resistance against *P. olseni* indicate there is a significant possibility of limiting the impact of this disease through genetic selection, not only in European clam aquaculture but also more broadly, as mass infestation and mortality due to this pathogen has also been frequently reported in Asia (87–90). That said, GxE interactions are known to be important factors in genetic selection trials, meaning that selection in a one environment may not prove effective in another. Given the presence of highly variable abiotic conditions in the Venice lagoon, coupled with the strong correlations between salinity/temperature and *P. olseni* infection intensity and prevalence, it is likely that selected families that perform best in this specific environment may not perform as well for any of the selected traits in another clam production are such as along the Asian coast.

## Conclusions

While the Manila clam is one of the most produced bivalve species in the world, the availability of genetic tools necessary to initiate selective breeding lag far behind those developed for most other cultured fish and shellfish. Here we showed that it is possible to design and carry out field challenges for one of the major clam pathogens, simulating commercial growing conditions. Likewise, we demonstrated that using mass spawning, the routine method for reproduction of clams in hatcheries, yields a sufficiently high number of full-sib families to reliably estimate genetic parameters and to provide a sufficient number of selection candidates. The development of a SNP panel allowed for partial parentage assignment, albeit further improvement in successful genotyping and assignment rate is required for greater efficiency and more accurate estimation of genetic parameters. The possibility of reconstructing pedigrees is important not only for estimating genetic parameters and managing inbreeding, but it will be required to use sib testing for measuring phenotypes that either cannot be recorded in selection candidates or could be risky to measure on them, as in the case of resistance to *P. olseni*. Being a chronic parasitic disease, directly challenging selection candidates might lead to introducing the parasite in the broodstock population, while sib testing would allow to maintain a *Perkinsus*-free broodstock. In turn, this should guarantee the production of parasite-free spat.

Finally, these preliminary results indicate that the high heritability for resistance against perkinsosis and the apparent lack of genetic correlation with growth-traits hold the promise to mitigate the effects of this climate-related parasitic disease without affecting growth performance.

## Data Availability Statement

The dataset can be found at: https://doi.org/10.5061/dryad.rr4xgxd6d.

## Author Contributions

MS, CP, LB, and FE designed the experiment. EV and J-FA produced the experimental population. GA and GD carried out the Perkinsus analysis. SF and MS contributed to the bioinformatic analysis. LG and RM carried out the genotyping. FE, MS, and PH carried the quantitive genetic analysis. MS and LB drafted the manuscript. All authors contributed comments to the draft.

## Conflict of Interest

The authors declare that the research was conducted in the absence of any commercial or financial relationships that could be construed as a potential conflict of interest.

## References

[1] Stentiford GD Sritunyalucksana K Flegel TW Williams BAP Withyachumnarnkul B Itsathitphaisarn O. New paradigms to help solve the global aquaculture disease crisis. PLoS Pathog. (2017) 13:e1006160. 10.1371/journal.ppat.1006160 28152043 PMC5289612

[2] World Bank Reducing Disease Risks in Aquaculture. World Bank Report #88257-GLB (2014).

[3] Rowley AF Cross ME Culloty SC Lynch SA Mackenzie CL Morgan E The potential impact of climate change on the infectious diseases of commercially important shellfish populations in the Irish Sea—a review. ICES J Mar Sci. (2014) 71:741–59. 10.1093/icesjms/fst234

[4] Tan K Zhang H Zheng H Selective breeding of edible bivalves and its implication of global climate change. Rev Aquac. (2020) 1–14. 10.1111/raq.12458

[5] Cohen RE James CC Lee A Martinelli MM Muraoka WT Ortega M Marine host-pathogen dynamics: influences of global climate change. Oceanography. (2018) 31:182–93. 10.5670/oceanog.2018.201

[6] Bishop SC Disease resistance: genetics. Encycl Anim Sci CABI. (2010) 288–90. 10.1081/E-EAS120019566

[7] Cock J Gitterle T Salazar M Rye M Breeding for disease resistance of Penaeid shrimps. Aquaculture. (2009) 286:1–11. 10.1016/j.aquaculture.2008.09.011

[8] Ødegård J Baranski M Gjerde B Gjedrem T Methodology for genetic evaluation of disease resistance in aquaculture species: challenges and future prospects. Aquac Res. (2011) 42:103–14. 10.1111/j.1365-2109.2010.02669.x

[9] Hollenbeck CM Johnston IA. Genomic tools and selective breeding in molluscs. Front Genet. (2018) 9:253. 10.3389/fgene.2018.00253 30073016 PMC6058216

[10] Guo H Zeng Q Li Y Wang Y Chen Z Lin P Genomic tools and selective breeding in molluscs estimating realized heritability for growth in zhikong scallop (Chlamys farreri) using genome-wide complex trait analysis. Aquaculture. (2018) 497:103–8. 10.1016/j.aquaculture.2018.07.046

[11] Barros J Velasco LA Winkler FM Heritability, genetic correlations and genotype by environment interactions in productive traits of the caribbean scallop, argopecten nucleus (Mollusca: Bivalvia). Aquaculture. (2018) 488:39–48. 10.1016/j.aquaculture.2018.01.011

[12] Ibarra AM Ramirez JL Ruiz CA Cruz P Avila S Realized heritabilities and genetic correlation after dual selection for total weight and shell width in catarina scallop (Argopecten ventricosus). Aquaculture. (1999) 175:227–41. 10.1016/S0044-8486(99)00100-3

[13] Liang B Jiang F Zhang S Yue X Wang H Liu B Genetic variation in vibrio resistance in the clam meretrix petechialis under the challenge of vibrio parahaemolyticus. Aquaculture. (2017) 468:458–63. 10.1016/j.aquaculture.2016.10.037

[14] Zhang A Wang L Yang X Hu X Fu Y Li C Relationship between shell morphological traits and body weight in two estuarine clams, meretrix meretrix and cyclina sinensis in shuangtaizi estuary, bohai sea in China. J Shellfish Res. (2018) 37:989 10.2983/035.037.0509

[15] Bai Z Li Q Han X Li J Estimates of genetic parameters and genotype by environment interactions for shell nacre color and growth traits in the purple freshwater pearl mussel Hyriopsis cumingii. Aquac Int. (2017) 25:2079–90. 10.1007/s10499-017-0170-x

[16] He M Guan Y Yuan T Zhang H Realized heritability and response to selection for shell height in the pearl oyster *Pinctada fucata* (Gould). Aquac Res. (2008) 39:801–5. 10.1111/j.1365-2109.2008.01889.x

[17] Lemer S Saulnier D Gueguen Y Planes S. Identification of genes associated with shell color in the black-lipped pearl oyster, *Pinctada margaritifera*. BMC Genomics. (2015) 16:568. 10.1186/s12864-015-1776-x 26231360 PMC4521380

[18] Jerry DR Kvingedal R Lind CE Evans BS Taylor JJU Safari AE Donor-oyster derived heritability estimates and the effect of genotype × environment interaction on the production of pearl quality traits in the silver-lip pearl oyster, *Pinctada maxima*. Aquaculture. (2012) 338–41:66–71. 10.1016/j.aquaculture.2012.02.001

[19] Bonneuil C Thomas F Recherche publique et régimes de production des savoirs de Mendel aux OGM. Gènes Pouvoirs et Profits. (2009).

[20] Gjedrem T Genetic improvement for the development of efficient global aquaculture: a personal opinion review. Aquaculture. (2012) 344–49:12–22. 10.1016/j.aquaculture.2012.03.003

[21] Gjedrem T Rye M Selection response in fish and shellfish: a review. Rev Aquac. (2018) 10:168–79. 10.1111/raq.12154

[22] Elaswad A Dunham R Disease reduction in aquaculture with genetic and genomic technology: current and future approaches. Rev Aquac. (2018) 10:876–98. 10.1111/raq.12205

[23] Gjedrem T Disease resistant fish and shellfish are within reach: a review. J Mar Sci Eng. (2015) 3:146–53. 10.3390/jmse3010146

[24] Yáñez JM Newman S Houston RD. Genomics in aquaculture to better understand species biology and accelerate genetic progress. Front Genet. (2015) 6:128. 10.3389/fgene.2015.00128 25883603 PMC4381651

[25] Vandeputte M Haffray P. Parentage assignment with genomic markers: a major advance for understanding and exploiting genetic variation of quantitative traits in farmed aquatic animals. Front Genet. (2014) 5:432. 10.3389/fgene.2014.00432 25566319 PMC4264515

[26] Lapègue S Harrang E Heurtebise S Flahauw E Donnadieu C Gayral P. Development of SNP-genotyping arrays in two shellfish species. Mol Ecol Resour. (2014) 14:820–30. 10.1111/1755-0998.12230 24447767

[27] Nguyen TTT Hayes BJ Ingram BA Genetic parameters and response to selection in blue mussel (*Mytilus galloprovincialis*) using a SNP-based pedigree. Aquaculture. (2014) 420–1:295–301. 10.1016/j.aquaculture.2013.11.021

[28] Liu T Li Q Kong L Yu H Comparison of microsatellites and SNPs for pedigree analysis in the Pacific oyster Crassostrea gigas. Aquac Int. (2017) 25:1507–19. 10.1007/s10499-017-0127-0

[29] Harney E Lachambre S Roussel S Huchette S Enez F Morvezen R Transcriptome based SNP discovery and validation for parentage assignment in hatchery progeny of the European abalone Haliotis tuberculata. Aquaculture. (2018) 491:105–13. 10.1016/j.aquaculture.2018.03.006

[30] Gosling E. Marine Bivalve Molluscs. Hoboken, NJ: Wiley Blackwell (2015).

[31] FAO The State of World Fisheries and Aquaculture 2018: Meeting the Sustainable Development Goals. Rome: FAO (2018).

[32] FAO FAO Yearbook. Fishery and Aquaculture Statistics 2014, United Nations, Text. Rome: FAO (2016). p. 76.

[33] Flassch J Leborgne Y Introduction in Europe, from 1972 to 1980, of the Japanese Manila clam. ICES Mar Sci Symp. (1992) 194:92–6.

[34] Zhao L Yan X Huo Z Yang F Zhang G Divergent selection for shell length in the Manila clam, *Ruditapes philippinarum*. J. World Aquac Soc. (2012) 43:878–84. 10.1111/j.1749-7345.2012.00612.x

[35] Huo Z Li Y Zhang X Yan X Yang F Growth improvement of shell length in the orange strain of Manila clam, *Ruditapes philippinarum*. J World Aquac Soc. (2017) 48:860–6. 10.1111/jwas.12392

[36] Yan X Huo Z Yang F Zhang G Heritability of larval and juvenile growth for two stocks of Manila clam *Ruditapes philippinarum*. Aquac Res. (2014) 45:484–90. 10.1111/j.1365-2109.2012.03250.x

[37] Trinkler N Sinquin G Querne J Paillard C. Resistance to brown ring disease in the Manila clam, *Ruditapes philippinarum*: a study of selected stocks showing a recovery process by shell repair. J Invertebr Pathol. (2010) 104:8–16. 10.1016/j.jip.2009.12.007 20035765

[38] Wang H Chai X Liu B Estimation of genetic parameters for growth traits in cultured clam Meretrix meretrix (Bivalvia: Veneridae) using the Bayesian method based on Gibbs sampling. Aquac Res. (2011) 42:240–7. 10.1111/j.1365-2109.2010.02617.x

[39] Wang C Huan P Yue X Yan M Liu B. Molecular characterization of a glutathione peroxidase gene and its expression in the selected Vibrio-resistant population of the clam Meretrix meretrix. Fish Shellfish Immunol. (2011) 30:1294–302. 10.1016/j.fsi.2011.03.015 21440068

[40] Yue X Wang H Huang X Wang C Chai X Wang C. Single nucleotide polymorphisms in i-type lysozyme gene and their correlation with vibrio-resistance and growth of clam Meretrix meretrix based on the selected resistance stocks. Fish Shellfish Immunol. (2012) 33:559–68. 10.1016/j.fsi.2012.06.007 22728564

[41] Proestou DA Vinyard BT Corbett RJ Piesz J Allen SK Small JM Performance of selectively-bred lines of eastern oyster, *Crassostrea virginica*, across eastern US estuaries. Aquaculture. (2016) 464:17–27. 10.1016/j.aquaculture.2016.06.012

[42] Frank-Lawale A Allen SK Dégremont L Breeding and domestication of eastern oyster (*Crassostrea virginica*) Lines for Culture in the Mid-Atlantic, USA: line development and mass selection for disease resistance. J Shellfish Res. (2014) 33:153–65. 10.2983/035.033.0115

[43] Gaffney PM Bushek D Genetic aspects of disease resistance in oysters. J Shellfish Res. (1996) 15:135–140.

[44] Bushek D Allen SK Host-parasite interactions among broadly distributed populations of the eastern oyster *Crassostrea virginica* and the protozoan Perkinsus marinus. Mar Ecol Prog Ser. (1996) 139:127–41. 10.3354/meps139127

[45] Pretto T Zambon M Civettini M Caburlotto G Boffo L Rossetti E Massive mortality in Manila clams (*Ruditapes philippinarum*) farmed in the Lagoon of Venice, caused by Perkinsus olseni. Bull Eur Assoc Fish Pathol. (2014) 34:43–53.

[46] Shimokawa J Yoshinaga T Ogawa K. Experimental evaluation of the pathogenicity of Perkinsus olseni in juvenile Manila clams *Ruditapes philippinarum*. J Invertebr Pathol. (2010) 105:347–51. 10.1016/j.jip.2010.08.007 20807538

[47] Il Park K. Figueras A Choi KS Application of enzyme-linked immunosorbent assay (ELISA) for the study of reproduction in the Manila clam *Ruditapes philippinarum* (Mollusca: Bivalvia): II. Impacts of Perkinsus olseni on clam reproduction. Aquaculture. (2006) 251:182–91. 10.1016/j.aquaculture.2005.06.003

[48] Jasim Uddin M Yang HS Choi KS Kim HJ Hong JS Cho M Seasonal changes in Perkinsus olseni infection and gametogenesis in manila clam, *Ruditapes philippinarum*, from Seonjaedo Island in Incheon, off the west coast of Korea. J World Aquac Soc. (2010) 41:93–101. 10.1111/j.1749-7345.2009.00337.x

[49] Casas SM Villalba A Reece KS. Study of perkinsosis in the carpet shell clam Tapes decussatus in Galicia (NW Spain). I. Identification of the aetiological agent and *in vitro* modulation of zoosporulation by temperature and salinity. Dis Aquat Organ. (2002) 50:51–65. 10.3354/dao050051 12152905

[50] Umeda K Shimokawa J Yoshinaga T Effects of temperature and salinity on the *in vitro* proliferation of trophozoites and the development of zoosporangia in Perkinsus olseni and honshuensis P, both infecting. Fish Pathol. (2013) 48:13–6. 10.3147/jsfp.48.13

[51] Drinkwater KF Beaugrand G Kaeriyama M Kim S Ottersen G Perry RI On the processes linking climate to ecosystem changes. J Mar Syst. (2010) 79:374–88. 10.1016/j.jmarsys.2008.12.014

[52] Pranovi F Caccin A Franzoi P Malavasi S Zucchetta M Torricelli P. Vulnerability of artisanal fisheries to climate change in the Venice Lagoona. J Fish Biol. (2013) 83:847–64. 10.1111/jfb.12124 24090551

[53] Burge CA Mark Eakin C Friedman CS Froelich B Hershberger PK Hofmann EE. Climate change influences on marine infectious diseases: implications for management and society. Ann Rev Mar Sci. (2014) 6:249–77. 10.1146/annurev-marine-010213-135029 23808894

[54] Grigorovich IA Korniushin AV MacIsaac HJ Moitessier's pea clam Pisidium moitessierianum (Bivalvia, Sphaeriidae): a cryptogenic mollusc in the Great Lakes. Hydrobiologia. (2000) 435:153–65. 10.1023/A:1004066609445

[55] Chapuis H Vandeputte M Dupont-Nivet M Haffray P Quillet E Selection for an improved disease resistance using factorial mating designs and molecular based pedigree in fish: a simulation study. In: 9th World Congress on Genetics Applied to Livestock Production. (2010). Available online at: https://hal.archives-ouvertes.fr/hal-01193740/document (accessed September 20, 2020).

[56] Haffray P Enez F Bugeon J Chapuis H Dupont-Nivet M Chatain B Accuracy of BLUP breeding values in a factorial mating design with mixed families and marker-based parentage assignment in rainbow trout *Oncorhynchus mykiss*. Aquaculture. (2018) 490:350–4. 10.1016/j.aquaculture.2018.03.003

[57] Dupont-Nivet M Vandeputte M Haffray P Chevassus B Effect of different mating designs on inbreeding, genetic variance and response to selection when applying individual selection in fish breeding programs. Aquaculture. (2006) 252:161–70. 10.1016/j.aquaculture.2005.07.005

[58] Ríos R Aranguren R Gastaldelli M Arcangeli G Novoa B Figueras A. Development and validation of a specific real-time PCR assay for the detection of the parasite Perkinsus olseni. J Invertebr Pathol. (2020) 169:107301. 10.1016/j.jip.2019.107301 31794707

[59] Ghiselli F Komissarov A Milani L Dunham J Breton S Nuzhdin S The draft genome of *Ruditapes philippinarum* (the Manila clam), a promising model system for mitochondrial biology. PeerJ. (2017). 10.7287/peerj.preprints.3096

[60] Kimura M Crow JF The measurement of effective population number. Evolution. (1963) 17:279–88. 10.2307/2406157

[61] Chevassus B Aspects génétiques de la constitution de populations d'élevage destinées au repeuplement. Bull Français La Pêche La Piscic. (1989) 314:146–68. 10.1051/kmae:1989010

[62] Falconer DS. Introduction to Quantitative Genetics. New York, NY: Ronald Press Co (1960).

[63] Lynch M Walsh B Genetics and Analysis of Quantitative Traits. Sunderland: Sinauer (2002).

[64] Azéma P Lamy J-B Boudry P Renault T Travers M-A Dégremont L. Genetic parameters of resistance to Vibrio aestuarianus, and OsHV-1 infections in the Pacific oyster, *Crassostrea gigas*, at three different life stages. Genet Sel Evol. (2017) 49:23. 10.1186/s12711-017-0297-2 28201985 PMC5311879

[65] Froehlich HE Gentry RR Halpern BS. Global change in marine aquaculture production potential under climate change. Nat Ecol Evol. (2018) 2:1745–50. 10.1038/s41559-018-0669-1 30201967

[66] de Lorgeril J Lucasson A Petton B Toulza E Montagnani C Clerissi C. Immune-suppression by OsHV-1 viral infection causes fatal bacteraemia in Pacific oysters. Nat Commun. (2018) 9:4215. 10.1038/s41467-018-06659-3 30310074 PMC6182001

[67] Jones GG Sanford CL Jones BL Manila Clams : Hatchery and Nursery Methods. (1993). Available online at: http://innovativeaqua.com/Publication/clam.pdf (accessed December 5, 2017).

[68] Toba D Small-Scale Clam Farming for Pleasure and Profit in Washington. Seattle, WA: Washington Sea Grant Program Publications (2005).

[69] Zhang G Yan X A new three-phase culture method for Manila clam, *Ruditapes philippinarum*, farming in northern China. Aquaculture. (2006) 258:452–61. 10.1016/j.aquaculture.2006.04.046

[70] Villalba A Reece KS Camino Ordás M Casas SM Figueras A Perkinsosis in molluscs: a review. Aquat Living Resour. (2004) 17:411–32. 10.1051/alr

[71] Waki T Yoshinaga T Experimental challenges of juvenile and adult Manila clams with the protozoan Perkinsus olseni at different temperatures. Fish Sci. (2013) 79:779–86. 10.1007/s12562-013-0651-4

[72] Villalba A Casas SM López C Carballal MJ. Study of perkinsosis in the carpet shell clam Tapes decussatus in Galicia (NW Spain). II. Temporal pattern of disease dynamics and association with clam mortality. Dis Aquat Organ. (2005) 65:257–67. 10.3354/dao065257 16119895

[73] Waki T Yoshinaga T Suppressive effects of low salinity and low temperature on in-vivo propagation of the protozoan Perkinsus olseni in Manila clam. Fish Pathol. (2015) 50:16–22. 10.3147/jsfp.50.16

[74] Gutierrez AP Turner F Gharbi K Talbot R Lowe NR Peñaloza C. Development of a medium density combined-species SNP array for pacific and european oysters (*Crassostrea gigas* and Ostrea edulis). G3 Genes Genom Genet. (2017) 7:2209–18. 10.1534/g3.117.041780 28533337 PMC5499128

[75] Enez F Morvazen R Lamy J-B Dégrémont L Guéméné D Mahla R Mass selection with factorial mating designs and DNA-parentage assignment is usable to improve survival to summer mortality in Pacific oyster *Crassostrea gigas*, AQUA. (2018). Montpellie (n.d.).

[76] Li R Qi L Yu R Parentage determination and effective population size estimation in mass spawning pacific oyster, Crassostrea gigas, based on microsatellite analysis. J World Aquac Soc. (2009) 40:667–77. 10.1111/j.1749-7345.2009.00286.x

[77] Zenger KR Khatkar MS Jones DB Khalilisamani N Jerry DR Raadsma HW. Genomic selection in aquaculture: application, limitations and opportunities with special reference to marine shrimp and pearl oysters. Front Genet. (2019) 10:693. 10.3389/fgene.2018.00693 30728827 PMC6351666

[78] Gjedrem T Selection and Breeding Programs in Aquaculture. New York, NY: Springer (2005).

[79] Pante MJR Gjerde B McMillan I Effect of inbreeding on body weight at harvest in rainbow trout, *Oncorhynchus mykiss*. Aquaculture. (2001) 192:201–11. 10.1016/S0044-8486(00)00467-1

[80] Knibb W Whatmore P Lamont R Quinn J Powell D Elizur A Can genetic diversity be maintained in long term mass selected populations without pedigree information?—a case study using banana shrimp Fenneropenaeus merguiensis. Aquaculture. (2014) 428–9:71–8. 10.1016/j.aquaculture.2014.02.026

[81] Tave D Inbreeding and Broodstock Management. 392nd ed Rome, Italy: FAO (1999).

[82] FAO FAO Technical Guidelines for Responsible Fisheries 5 Suppl 3 Aquaculture Development 3. Genetic resource management (2008).

[83] Sae-Lim P Kause A Mulder HA Olesen I. BREEDING AND GENETICS SYMPOSIUM: climate change and selective breeding in aquaculture. J Anim Sci. (2017) 95:1801–12. 10.2527/jas.2016.1066 28464113

[84] Noble TH Coman GJ Wade NM Thomson PC Raadsma HW Khatkar MS Genetic parameters for tolerance to gill-associated virus under challenge-test conditions in the black tiger shrimp (*Penaeus monodon*). Aquaculture. (2020) 516:734428 10.1016/j.aquaculture.2019.734428

[85] Huber K. Invited review: resource allocation mismatch as pathway to disproportionate growth in farm animals—prerequisite for a disturbed health. Animal. (2018) 12:528–36. 10.1017/S1751731117002051 28803599

[86] Hocking PM. Unexpected consequences of genetic selection in broilers and turkeys: problems and solutions. Br Poult Sci. (2014) 55:1–12. 10.1080/00071668.2014.877692 24397366

[87] Yang HS Park KJ Choi KS Pathologic survey on the Manila clam *Ruditapes philippinarum* (Adams and Reeve 1850) from haeju off the western coastal yellow sea. Ocean Sci J. (2010) 45:93–100. 10.1007/s12601-010-0008-1

[88] Xie Z Xie L Fan Q Pang Y Deng X Xie ZQ. A duplex quantitative real-time PCR assay for the detection of Haplosporidium and Perkinsus species in shellfish. Parasitol Res. (2013) 112:1597–606. 10.1007/s00436-013-3315-5 23371501

[89] Choi K-S Waki T Perkinsus olseni (Lester and Davis 1981) infection in the Manila clam (*Ruditapes philippinarum*) in Korea: species identification, impacts and spatio-temporal distribution. Bull Japan Fish Res Educ Agency. (2016) 42:23–27.

[90] Nam KW Do Jeung H Song JH Park KH Choi KS Il Park K. High parasite burden increases the surfacing and mortality of the Manila clam (*Ruditapes philippinarum*) in intertidal sandy mudflats on the west coast of Korea during hot summer. Parasites Vectors. (2018) 11:42. 10.1186/s13071-018-2620-3 29347957 PMC5774171

