# Supplementary material for: Potential for Genetic Improvement of Resistance to Perkinsus olseni in the Manila Clam, Ruditapes philippinarum, Using DNA Parentage Assignment and Mass Spawning
Source: Front Vet Sci. 2020 Oct 22;7:579840. doi: 10.3389/fvets.2020.579840 (PMC7649815; doi:10.3389/fvets.2020.579840)
Supplement: Supplementary file 1 [file Data_Sheet_1.docx]

**Comparative DNA extractions:**

Comparison of DNA concentration for extractions performed on gill and mantle tissue of the same *R. philippinarum* individuals (results provided by Labogena, Jouy-en-Josas, France) (Table a). ANOVA test (Table b) shows that there is a significant difference in the DNA concentration, with gill tissue providing a higher concentration than mantle tissue.

Table a: DNA concentrations from extractions performed at Labogena, using gill and mantle tissue from the same individuals and using the same extraction method.

| **DNA ng/uL** | **Tissue** | |
| --- | --- | --- |
| Individual | Gill | Mantle |
| Clam 1 | 97.09 | 72.63 |
| Clam 2 | 91.15 | 47.24 |
| Clam 3 | 141.21 | 56.79 |
| Clam 4 | 127.12 | 51.26 |
| Clam 5 | 141.23 | 48.63 |
| Clam 6 | 125.54 | 64.44 |
| Clam 7 | 142.9 | 62.06 |
| Clam 8 | 124.33 | 70.48 |
| Clam 9 | 75.26 | 47.33 |
| Clam 10 | 146.36 | 67.43 |
| Clam 11 | 129.91 | 50.24 |
| Clam 12 | 109.62 | 85.94 |
| Clam 13 | 112.96 | 70.34 |
| Clam 14 | 152.77 | 81.82 |
| Clam 15 | 105.82 | 70.87 |

Table b: ANOVA test results showing a significant difference between gill and mantle tissue for the DNA concentration.

| **Result Details One-Way Repeated Measures ANOVA** | | | | |
| --- | --- | --- | --- | --- |
| Source | SS | df | MS |  |
| Between-treatments | 25565.7698 | 1 | 25565.77 | F = 89.3778 |
| Within-treatments | 9279.9235 | 28 | 331.4258 | p-val < 0.05 |
| Error | 4004.5824 | 14 | 286.0416 |  |

**Sex ratio of final sampling event:**

Sex, as a qualitative trait, could be determined for 890 individuals out of 1059 (Figure 2). 169 individuals were indeed not mature enough for sex determination. Final male to female ratio was 47:53.

Figure 2: Sex distribution of Chioggia experimental F1 clams (n=1059).

**Genetic gain comparisons for shell weight (SW) and total weight (TW):**

The Falconer (1960) formula to calculate expected response to selection of a trait (*R*):

$$R_{trait a}=i\times h_{a}^{2}\times\sigma_{a}$$

$i$ $=intensity of selection pressure$

$$h_{a}^{2}=estimated heritability of trait a$$

$$\sigma_{a}=phenotypic variability of trait a$$

The formula is adapted for a correlated response (i.e. the effect on trait b of selecting for trait a):

$$R_{trait b}=i\times\sqrt{h_{a}^{2}}\times\sqrt{h_{b}^{2}}\times r_{A}\times\sigma_{b}$$

$$r_{A}=genetic correlation between traits a and b$$

$$h_{b}^{2}=estimated heritability of trait b$$

$$\sigma_{b}=phenotypic variability of trait b$$

Below, the data for SW and TW are shown, as well as the value of 5% selection pressure as an example, though this parameter has no effect on the comparison in genetic gain between the two selection strategies:

| Parameter | Value |
| --- | --- |
| $i_{5\%}$ | 2.063 |
| $h_{TW}^{2}$ | 0.23 |
| $h_{SW}^{2}$ | 0.35 |
| $r_{A}$ | 0.97 |
| $\sigma_{TW}$ | 3.63 |
| $\sigma_{SW}$ | 2.09 |

For direct selection of TW, expected response to selection would be +1.722 g per generation. If, however, selection were carried out for SW, then the correlated response on TW would mean an expected response of +2.061 g per generation. This represents a difference of 0.339 (19.7%) in gain per generation between the two selection methods.
